# Elastic Recovery In-Die During Cyclic Loading of Solid Anaerobic Digestate

**DOI:** 10.3390/ma17235976

**Published:** 2024-12-06

**Authors:** Grzegorz Łysiak, Ryszard Kulig

**Affiliations:** Department of Food Engineering and Machines, University of Life Sciences in Lublin, Głęboka St. 28, 20-612 Lublin, Poland

**Keywords:** anaerobic digestate, densification, compression, cyclic loading, elastic recovery, springback

## Abstract

Anaerobic digestate represents a valuable organic by-product, with one of the main challenges being its enhanced utilization. Pelletization offers potential benefits by improving the digestate’s storability, facilitating transport, and significantly expanding its application as a fertilizer or biofuel. Understanding the mechanisms of densification and their impact on the final product quality is essential and served as the inspiration for this research. Its primary focus was stress relaxation and the subsequent elongation of pellets within the compaction chamber (in-die). It investigated the hypothesis that elastic recovery, resulting from internal stress relaxation once the compressive force is removed, has direct implications for pellet quality. The investigations were conducted using a Zwick universal machine. Samples of digestate with varied moisture levels, i.e., 10, 13, 16, 19, and 22%, were loaded with amplitudes of 8, 11, 14, 17, and 20 kN. Ten loading and unloading cycles were employed. Elastic recovery (in-die) (ER_in-die_) in the investigated digestate increased with rising MC and compaction pressure but decreased with increasing cycle number. There was little correlation between ER_in-die_ and pellet strength. Permanent strain energy exerted the greatest influence on pellet quality. Permanent strain energy had the greatest influence on pellet quality. Examining hysteresis loop behavior emerged as a promising area for further research to better understand springback phenomena.

## 1. Introduction

The concepts of circular economy and sustainable development present relatively recent but crucial challenges for modern industry and scientific research. These frameworks require innovative approaches to managing raw materials, energy, and waste, driving demand for novel products, new materials, and optimized technologies. This context introduces novel research opportunities and establishes a direction for future studies. Digestate, a by-product of biogas production, is one example of such a material. Effectively utilizing this material can improve the efficiency of biogas facilities and support the development of new products, including fertilizers [1,2], composites [3,4], and biofuels [5,6,7]. Approaches for effective digestate management are under investigation, with the preliminary findings summarized in the authors’ previous works [8,9]. Densifying digestate into pellet form is one value-added approach to its management. A thorough understanding of this material’s behavior during densification is essential for optimizing its applications and the related processing technologies.

Elastic recovery, springback, or expansion rate in the densification process relates to a compressed material’s ability to rebound and decrease in density when the applied pressure is withdrawn. This undesirable effect, which occurs frequently during biomass pelletization and briquetting, is critical to the processing performance in terms of pellet quality. The recoverable (elastic) deformation of compacted particles takes place in-die and out-of-die [10]. In the first scenario, it represents the internal reaction of the compacted material to external stresses caused by the piston, rollers, etc. In the second, after exiting the die, the pellet goes through the process of relaxing internal stresses over a significantly longer time. During this time, structural rearrangements occur, and the temperature or the moisture introduced in the process tends to equilibrate within the pellet and with the environment, and the pellet stretches, bends, cracks, or breaks [11].

The proportion of elastic and plastic deformations, breaking, or relaxation determines the elastic recovery mechanisms. Overall, the elastic recovery of biomass during densification is a complex interplay of various mechanisms and is influenced by several factors, including (1) material properties, e.g., moisture [12,13,14,15] and chemical composition [16,17], (2) the densification process parameters, e.g., pressure and pressure history [14,18,19], holding time [20], temperature [13], pre-processing operations, e.g., torrefaction or the addition of binders [21], and (3) the storage conditions of the final product [12,22,23].

The effect of compaction pressure and moisture content on elastic springback was shown to differ amongst bioresidues [14]. The elongation of wood pellets was demonstrated to decrease with increasing moisture levels [12]. Trinh et al. [24] reported lower springback at increasing pressure values of beech biomass. In contrast, Bashaiwoldu et al. [25] found greater values of volumetric elastic recovery for microcrystalline cellulose pellets at higher compaction pressures. In turn, Wongsiriamnuay et al. [26] linked the presence of the impact to pressure magnitude. The study indicated no significant difference in elastic recovery for pellets from maize residues under pressures ranging from 150 to 250 MPa. According to Granado et al. [27] briquettes of cassava waste generated under lower pressure showed the greatest volume variance. According to Tumuluru et al. [28], for pellets of most feeds, the maximum elastic recovery occurs shortly after pressure release and declines with time. For wood pellets, the fastest elongation occurred within the first 2 or 3 min [12].

From a practical standpoint, much of the research on densification focuses on the out-of-die impact [29]. Significant dimensional changes in the pellets may occur during this time, which begins as soon as they exit the die and can persist for up to a week or longer [16,30]. They may be easily linked to the overall quality of finished items. Elastic recovery can alter pellet density and durability [31].

Haware et al. demonstrated [32] that the elastic recovery in-die can be estimated experimentally and used to predict the tabletability of diverse pharmaceutical materials. More importantly, elastic recovery was discovered to have a linear relationship with out-of-die elasticity characteristics. Ilić et al. [31,33] stated that the main difference between the in-die and out-of-die methods is the inclusion or exclusion of the elasticity of the material studied.

The in-die profile incorporates elastic deformation since the tablet dimensions are derived under stress during compression when the tablet does not return the elastic energy stored inside it. In contrast, the out-of-die approach lacks this elastic feature, resulting in greater accuracy. According to Vreeman et al. [19,34], the in-die approach overestimates compressibility values, producing artificially higher compressibility than the out-of-die method. According to the authors, the difference between in-die and out-of-die values is due to the material’s flexibility. Furthermore, depending on the model used, considerable connections between the in-die and out-of-die effects were discovered [34].

The structural changes occurring during stress relaxation often lead to shape alterations or even the complete disintegration of the pellet, making direct measurement complex or infeasible. This has driven interest in using in-die ER measurements to avoid these difficulties and enable more automated data collection. Consequently, efforts focus on establishing correlations between in-die and out-of-die recovery or directly linking in-die deformation with pellet quality.

Recently, Łysiak et al. [8,9] evaluated recoverable and nonrecoverable responses occurring during confined axial compression of solid anaerobic digestate (SAD). Thanks to the method applied, they were able to evaluate the elastic response in-die, but no data on the springback effect have been provided [9]. This work verifies the hypothesis that elastic recovery in-die has direct implications for pellet quality, thus can be used to predict pelleting outcomes. Hence, the objective of this study was to examine the variance of elastic recovery in-die (ER_in-die_) as a function of compaction conditions (moisture content, pressure, and pressure history) and explore any correlations with densification parameters and pellet quality. There has been relatively little research on this topic for biomass residues, for which elastic recovery plays a practical and important role during their densification. In this regard, this study can help better understand the mechanisms occurring throughout the compaction processes.

## 2. Materials and Methods

### 2.1. Collection of Solid Anaerobic Digestate

Solid anaerobic digestate (SAD) was obtained from a biogas facility in Międzyrzec Podlaski owned by BioPower Sp. z o. o. (Zamość, Poland). The principal substrate used in the biogas plant was maize silage (70%), with a dry matter (DM) content of approximately 35%. The liquid substrate (30%) was molasses with 7% DM obtained from a local distillery.

### 2.2. Sample Preparation/Moisture Determination

A batch of SAD was ground with a hammer mill, applying a 3 mm mesh screen size. The initial moisture of the ground material was determined using the air-oven method. Three samples, weighing 5 g, were dried at 105 °C for 1 h according to the procedures described in PN-EN ISO 18134-3:2023-12 [35]. Five samples were obtained from the batch and acquired at different moisture levels, i.e., 10, 13, 16, 19, and 22% w.b. The range of moisture is used for pelleting a variety of biomaterials [36]. The added/removed water amount was calculated using simple mass balance equations. The samples were cooled for 24 h to equilibrate the water content.

### 2.3. Cyclic Loading/Unloading

A Zwick Z020/TN2S universal testing machine (Zwick GmbH, Ulm, Germany) was used in the experiments. A 2 g sample of digestate was placed in a closed die, 15 mm in diameter [8], and axially loaded to obtain the desired load level, either 8, 11, 14, 17, or 20 kN. The levels corresponded to the following pressure levels: 45.3, 62.3, 79.2, 96.2, and 113.2 MPa. When the initial load reached a value of 2 N, the force and displacement began to be recorded using Zwick’s testXpert software (version 7.11). The loading phase was followed by an unloading phase. When the unloading force reached the minimum of 2 N again, another loading/unloading cycle began. The cycle number was set to 10. During both loading and unloading, a constant compression rate of 10 mm min^−1^ was maintained. All the operating parameters were controlled using Zwick’s testXpert software. Data were collected from the software at a frequency of 10 Hz. For each moisture content and loading level, experiments were carried out in triplicate.

**Figure 1:**
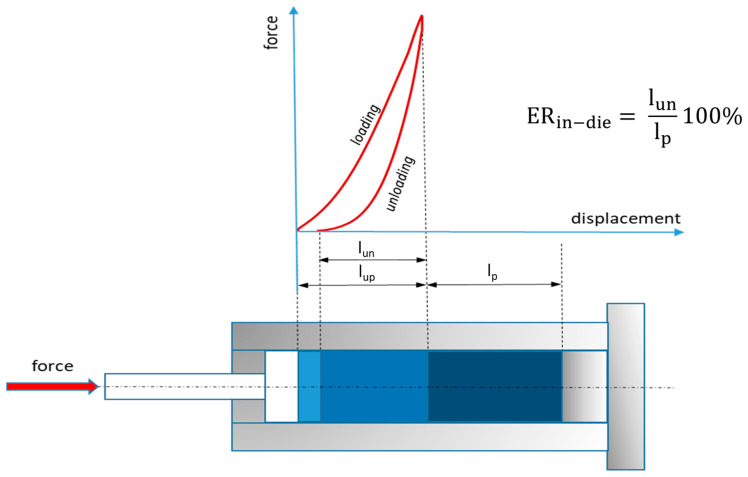
A schematic diagram for determining the in-die elastic response, ER_in-die_.

Based on the recorded load–displacement characteristics, values of head displacements, including total, permanent, and elastic values, for each loading cycle were determined. On the basis of these values, the elastic recovery ER_in-die_ (%) was calculated (Figure 1).

The recovery represents the relative increase in pellet length during the unloading stage [33,37] and was calculated using Equation (1):

(1) $${ER}_{in\text{-}die}=\frac{l_{un}}{l_{p}}\cdot 100\%$$

where pellet length (l_p_) denotes the height of the pellet at the end of each loading and unloading cycle. Thus, the elastic recovery of the material in-die was calculated for each of the ten cycles.

The elastic recovery after 24 h (ER_24_) was determined according to Equation (2):

(2) $${ER}_{24}=\frac{l_{24}-l_{p(10)}}{l_{p\left(10\right)}}\cdot 100\%$$

where l_24_ denotes the height of the pellet after 24 h of relaxation, and l_p(10)_ is the height of the pellet at the maximum pressure of the tenth cycle. Thus, ER_24_ is the arithmetic sum of ER_in-die_ and ER_out-of-die_. Due to the different approaches used in the literature, it should be noted that, in both cases, the pellet extension was related to the pellet height at maximum pressure.

For a deeper analysis of the springback nature, the compression energy and its distribution to the permanent energy (E_n-r_) and elastic (dissipated) (E_r_) energy were additionally calculated. The total and elastic energies represent the area under loading and unloading characteristics, respectively, but in the case of the non-recoverable part, this energy is represented by the area of the hysteresis loop [8].

### 2.4. Pellet Strength

As the result of ten loading/unloading cycles, compacted pellets were obtained. After the pellets left the compaction chamber, their dimensions were measured with a digital caliper (accuracy of 0.01 mm). The pellets were then stored for 24 h in a refrigerator. After the next 24 h, their dimensions were measured again. Subsequently, the strength of pellets was established using the Brazilian indirect tensile test, according to the procedures used in the literature on the subject [9,38,39]. The individual pellet was loaded perpendicular to its longitudinal axis using the Zwick universal machine. The compression rate was adjusted to 10 mm min^−1^. The ultimate load related to the pellet length was determined as the index of pellet strength (SI) according to the equation:

(3) $$SI=\frac{2F_{max}}{\pi dl_{24}}\;[MPa]$$

where F_max_ corresponds to the force at the ultimate fracture point, l_24_ is the pellet length after 24 h, and d is the pellet diameter [40,41].

Measurements were performed on each pellet obtained from each experiment.

### 2.5. Statistical Analysis

Statistical analyses were performed using Statistica 13 (StatSoft, Inc. STATISTICA). Multivariate analysis of variance (MANOVA) was used for comparing means and to estimate the effect of pressure (P), moisture content (MC) and cycle number (CN) on elastic recovery. For both variance and regression analyses, the level of significance was set to *p* < 0.05.

## 3. Results

### 3.1. Effects of Moisture, Pressure and Cycle Number

**Figure 2:**
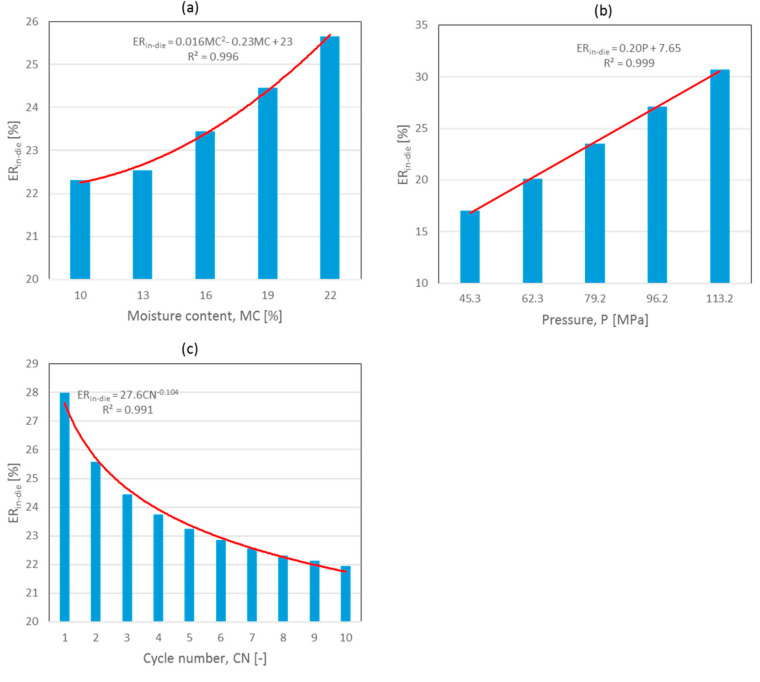
Effect of moisture content (**a**), applied pressure (**b**), and cycle number (**c**) of SAD on elastic recovery (ER_in-die_—elastic recovery, MC—moisture content, P—pressure, CN—cycle number).

The ER_in-die_ value increased with increasing digestate moisture content and applied pressure. However, it decreased with successive loading cycles. Mathematical functions with high determination coefficients (R^2^ > 0.991) were used to accurately describe the average changes in elastic recovery. Specifically, a quadratic function was used for moisture content, a linear function for the applied pressure, and a power function for the cycle number (Figure 2).

The results of the analysis of variance in the effects of moisture, pressure, and cycle number on the springback values (ER_in-die_) are shown in Table 1.

**Figure 3:**
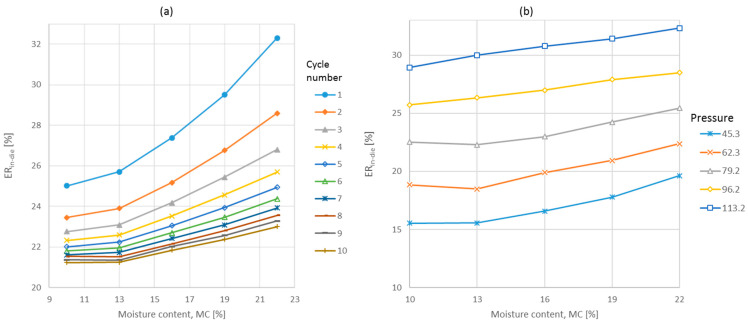
The interaction effects of moisture content with cycle number (**a**) and pressure (**b**) on the elastic recovery of SAD.

The largest source of variation was noted for the pressure applied, while the errors for moisture and cycle number were similar. All of the analyzed factors significantly influenced the elastic recovery magnitude. The interaction effects of moisture and pressure, and moisture and cycle number were also significant at *p* < 0.05 [8,9]. The results of these interactions are shown in Figure 3a,b. The most significant changes of ER_in-die_ occurred during the initial five cycles, while for lower moisture contents (10% and 13%) no or little effect was observed. However, a further increase in water content led to a closely linear increase in elastic recovery. With successive cycles, ER_in-die_ exhibited a declining trend that approached an asymptotic behavior. The magnitude of these declines decreased progressively as the cycle number advanced. The influence of moisture content, as depicted in Figure 2a and modeled by a quadratic function, exhibited a clear quadratic relationship at lower pressure values and during the initial loading cycles but displayed a more linear pattern at higher pressure values and throughout subsequent loading cycles.

Figure 3b displays the interaction between applied pressure values and moisture contents. While the dependence of ER_in-die_ on moisture content showed a polynomial of degree two for lower pressures (Figure 3b), it was clearly rectilinear for pressures above 79.2 MPa. On the other hand, the effect of applied pressure was rectilinear for all the studied moisture contents.

**Figure 4:**
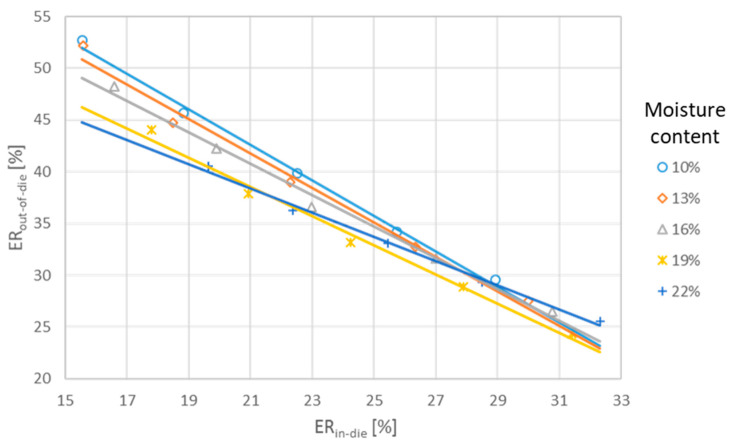
Relationships between elastic recovery in-die and out-of-die.

Based on the results of the current study, ER_in-die_ remained relatively constant for moisture contents of 10–13%. In the study presented in this paper, ER_in-die_ increased with increasing moisture contents and pressure values. Interestingly, the elastic recovery after 24 h decreased for pellet samples with increased moisture contents and subjected to increased pressure values. In the latter case, the results obtained are more consistent with those presented in the literature. This indicates that there is a negative correlation between elastic deformation inside and outside the die. This was confirmed by a deeper analysis of this study’s results. The closely linear correlation between ER_in-die_ and ER_out-of-die_ is presented in Figure 4.

An increase in SAD moisture content led to an increase in reversible strain ER_in-die_ and a decrease in the amount of strain recovery occurring outside the die. As a result, the total strain of *ER*_24_ slightly decreased as the moisture content of the digestate increased. Similarly, when pressure increased, the fraction of ER_in-die_ grew rectilinearly, whereas the strain of ER_out-of-die_ rectilinearly decreased. Consequently, *ER*_24_ decreased with increasing pressure values. The above shows that while comparing the numerous test results, it is critical to consider which components of the total elastic response are involved.

It is important to highlight the relatively high proportion of ER_in-die_ compared to the total ER_24_. In the current study, it varied from approximately 22% for MC of 10% and 45.3 MPa to 60% for MC of 22% and 113.2 MPa. Thus, the ER_in-die_ surpassed the relaxation values outside the die and ranged from approximately 30% for MC of 10% and 45.3 MPa to 126% for MC of 20% and 113.2 MPa [8,9].

### 3.2. Hysteresis Loops

**Figure 5:**
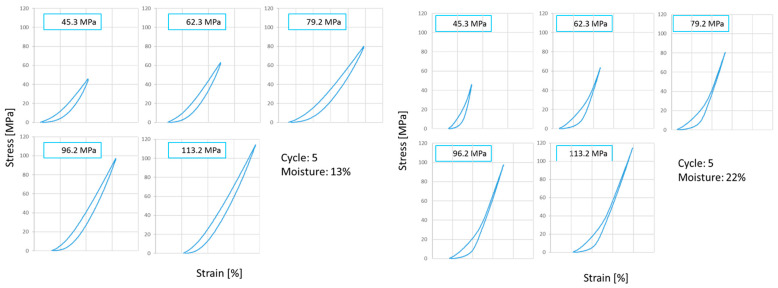
Hysteresis loops for 10 and 22% levels of moisture content of SAD.

To gain a better understanding of springback, previous studies were very helpful [8,9]. The authors clearly pointed out the differences in the course of loading/unloading curves depending on the moisture content. This relationship for the two extreme moisture levels is illustrated in Figure 5.

The most prominent observation is the quite easily distinguishable occurrence of the area of pure elastic response (PER) for a moisture content of 22%. For this moisture content, the pressure at which PER occurred was approximately 40–50 MPa. For digestate with the lowest moisture content, the establishment of a pressure value above which only PER occurs could not be objectively determined. The tendency was confirmed for intermediate moisture contents.

It must be noted that the percentage of elastic strain with respect to the total strain obtained during loading (l_un_/l_up_) increased (85.8–98.5%) in successive cycles. The presented hysteresis loops show that regardless of the moisture content, the entire strain was practically reversible (especially in subsequent loading cycles). This appears to indicate that the ER_in-die_ was predominantly dependent on the value of strain needed to reach a fixed pressure level. However, it must be noted that in the calculation of elastic in-die deformations, they are related to the length of the pellet (l_p_), which at the same time decreased with increasing pressure values and moisture contents. The percentage of the recovered strain grew with pressure of approximately 94–96.6% and decreased slightly with increasing moisture content (95.8–95.4%). However, at first glance, the above seems to disagree with the data presented in Figure 2, but it results from the mentioned differences in pellet length. It should also be noted that the effect of moisture content on springback values was less pronounced in further loading cycles (Figure 3) when the height of the pellets, due to successive loading, ceases to change significantly.

**Figure 6:**
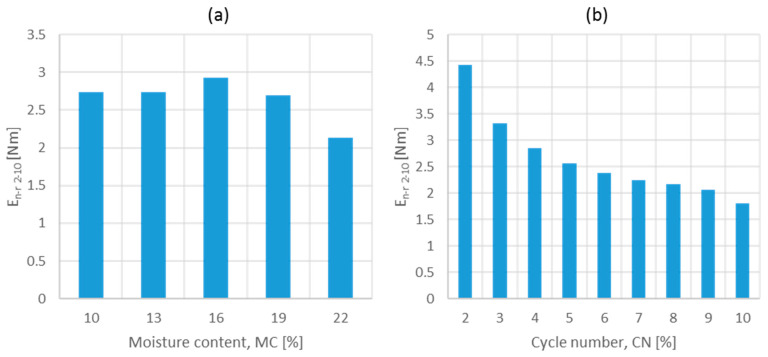
Effect of moisture content (**a**) and cycle number (**b**) on the area bounded between loading and unloading curves (without cycle 1).

Analysis of the strain hysteresis loops allowed for the identification of differences in the area bounded between the loading and deformation curves. This area decreased for moisture contents higher than 16% and asymptotically with an increasing number of cycles (Figure 6).

The greatest variation in this parameter was due to the increasing number of cycles and pressure values applied (Table 2).

### 3.3. Correlation Between Elastic Recovery and Pellet Strength

As mentioned earlier, elastic recovery is an important parameter, used to assess the quality of pellets and their susceptibility to shape preservation. The observations on the variability of elastic recovery in-die indicate its strong dependence on the cycle number. For a higher number of cycles, ER_in-die_ parameter seems less useful for assessing the impact of digestate moisture on its densification outcomes.

**Figure 7:**
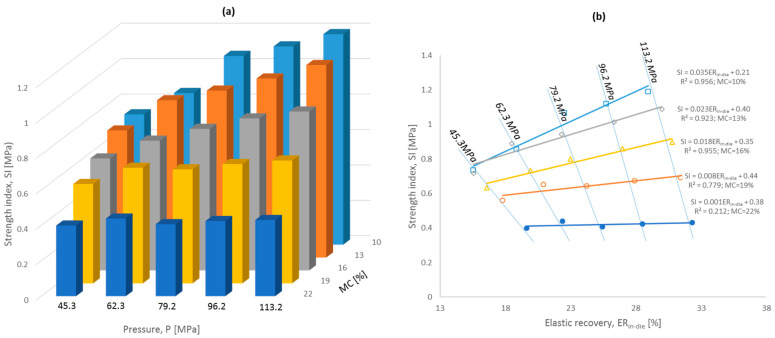
Strength index of pellets for variable pressure values and moisture contents (**a**), (**b**) effect of elastic recovery in-die ER_in-die_ on SI values.

Figure 7 shows the results of the strength characteristics of the pellets for the tested variable pressures and moisture contents of SAD. The SI index decreased for all of the applied pressures as the moisture content increased. The greatest variability occurred for the highest pressure applied. In contrast, the positive effect of the increasing pressure values on pellet strength was particularly pronounced for the lower moisture contents, and the pellet strength decreased above a moisture content of 16%. For a moisture content of 22%, the use of higher pressures, practically, did not result in any improvement in the strength characteristics of the pellets. It could also be observed that under identical conditions, springback was rectilinearly dependent on the pressure value and moisture content, with no discernible changes between the moisture levels. Furthermore, it should be highlighted that using higher pressures during compaction of digestate with a high moisture content did not considerably increase pellet strength [9].

The correlation between pellet strength and elastic recovery in-die ER_in-die_ (for 25 cases: MC × P) was positive and weak (0.141). The reason for this was the high variability in the applied pressure. Pellet strength slightly increased in successive loading cycles. On the other hand, when the different moisture levels were not considered, the strength of the pellets correlated with the springback (Figure 7b).

However, this was not the general rule, and the significance of the correlation decreased for high moisture contents. In this case, springback increased markedly for higher applied pressures; however, this was not reflected in higher pellet strength. It is important to note that elastic recovery in-die was positively correlated with pellet strength. Furthermore, a negative value could be expected, as greater expansion of the material and reduced density could possibly reduce the pellet strength. In contrast, for the same pressure level, an increase in ER_in-die_ due to a higher water content was accompanied by a decrease in pellet strength (higher springback, lower pellet strength). The graph demonstrates that pellet strength is more clearly related to changes in springback due to varied moisture content than to pressure values applied.

### 3.4. Correlation Between Elastic Recovery and Some Hysteresis Features

The hysteresis loops demonstrate (as described above, in Section 3.2) that with an increase in the number of cycles, the entire deformation was reversible. In contrast, as already noted in previous work, the shape of the hysteresis loop is intriguing. Since the hysteresis loop shape is also related to the size of the springback, a better understanding of springback required analyzing the correlation between springback and the energy inputs associated with permanent deformation and the energy dissipated during reversible deformation. In addition, the properties of the pellets (density and strength) were also considered. The results are shown in Table 3. For 25 cases (MC × P), the ER_in-die_ was highly correlated with pellet density (0.956). Higher values of ER_in-die_ were obtained for higher pellet densities. A strong correlation was evidenced between ER_in-die_ and energy dissipated (0.954) and a slightly weaker correlation was noted with a share of this energy. ER_in-die_ was also positively correlated with permanent strain energy (R = 0.752) and negatively with its percentage (−0.925). Higher springback values were determined for greater work losses. This can be explained by the authors’ previous observations, which indicate that the proportion of elastic energy increases with increasing moisture content. Pellet strength, on the other hand, was very weakly correlated with the analyzed parameters, except for the energy of permanent deformation (0.646). Moreover, this parameter was not significantly related to the share of this energy expenditure.

## 4. Discussion

The primary advantage of using in-die elastic recovery for pellet evaluation is its simplicity and automation potential, avoiding errors associated with measuring pellets after removal from the die. While post-die measurements are simple, they are less suitable for automated recording. Our research demonstrated a linear relationship between in-die and out-of-die recovery, consistent with similar dependencies observed by Mawla et al. [42] in studies on HPMC polymers. Katz and coworkers also calculated out-of-die compressibility based on in-die compressibility, taking both elastic and viscoelastic recovery into account [43,44]. They stated that their method for generating accurate out-of-die compressibility profiles is both universal and computationally simple. Hirschberg et al. [29] divided elastic recovery into in-die and out-of-die components, showing that in-die recovery was linearly dependent on compaction pressure, while out-of-die recovery depended on the material’s properties. The strong relationship observed between ER_in-die_ and ER_out-of-die_ in this study supports this finding.

The research findings offer only partial confirmation of the hypothesized relationship between in-die elastic recovery (ER) and pellet strength. This partial confirmation is based on two observations: (1) the relationship exists only under specific conditions, such as a constant load value, and (2) as material moisture content increases, the relationship between ER and pellet strength weakens and eventually disappears.

In this study’s design, which considered varying load levels and moisture contents, a general trend was observed where an increase in ER_in-die_ was accompanied by an increase in pellet strength. However, under constant pressure conditions, a significant negative correlation between ER and pellet strength emerged, suggesting that the relationship between these variables is complex and dependent on specific loading and moisture conditions.

The literature lacks consensus regarding the influence of pressure on the magnitude of elastic recovery. Styks et al. [14] noted an increase in ER for the higher moisture level of giant miscanthuss and Virginia mallow Sida but, conversely, a decrease of this parameter at higher pressure values. However, the measurement concerned a springback outside the die. The effect of pressure on elastic recovery of several pharmaceutical excipients was studied by Keizer et al. [45]. The authors observed increased and decreased springback with increasing pressure. Schönfeld et al. [46] for polymeric excipients noted a linear increase in ER with increasing pressure after a certain level of compaction. In that study, the authors indicated that exceeding a particle density threshold of polymeric excipients might be linked with the start of a linear increase in fast elastic recovery. They stated that the energy applied to the compact over a specific threshold value is converted into elastic deformation of the material itself. This energy is then released immediately during the unloading phase. The authors indicated that, for the predominantly brittle materials, an increase in compression pressure leads to an increase in the in-die elastic recovery. Cabiscol et al. [47] stated that ER for the studied pharmaceutics remains almost constant regardless of the compaction pressure. It must be noted that the above conclusions do not apply to the immediate springback in-die, but to a much longer relaxation time. In summary, the strong dependence of elastic recovery on experimental conditions significantly limits its usefulness for making general conclusions about the densification process or pellet quality.

Similarly to Schonfeld’s findings [46], where an increase in elastic deformation was attributed to reaching a pressure threshold beyond which energy is used exclusively for elastic deformation, our results show a noticeable decrease in this threshold pressure for higher-moisture material, above which pure elastic response occurs. This observation is supported by the hysteresis loops for dry and moist digestate, with the latter showing a distinct immediate elastic response once a critical pressure level is exceeded—a phenomenon not observed in dry material. It is plausible that further loading, though unnecessary in our case since the applied pressures were sufficient to produce durable pellets, might reveal this effect more clearly. In this context, we refer to the reaction of incompressible water. However, this explanation requires additional research. The current literature does not provide a definitive answer regarding the effect of moisture on springback magnitude. Dhamodaran and Afzal [20] observed higher “green” springback for higher moisture levels of white spruce softwood and maple hardwood pellets. Similarly, Hernandez et al. [48] reported higher ER for higher moisture contents of milled pine pellets. On the contrary, Saha et al. [15] observed that the ER of milled corn stover pellets was lower for higher moisture contents. In the authors’ previous studies [8,9], the elastic deformation was found to increase slightly with increasing moisture contents for the first loading cycle, while it was relatively constant in subsequent cycles. The elastic deformation also increased with increasing pressure values and decreased in subsequent cycles. Additionally, a very small decrease in elastic energy with a decrease in moisture content was observed. This energy did not change significantly between the cycles. By associating elastic deformation to pellet height, the elastic recovery ER_in-die_ was determined. It proved to be dependent on all three factors, i.e., moisture content, pressure, and cycle number.

## 5. Conclusions

The findings suggest that the hypothesized relationship between in-die elastic recovery and pellet quality is only partially supported. A positive linear relationship was found between in-die elastic recovery and pellet strength. However, as digestate moisture content increased, this effect diminished, becoming statistically insignificant at 22% moisture. While in-die elastic recovery showed high correlations with permanent or elastic strain energies and pellet density, its correlation with pellet strength across five moisture and pressure levels was very low. Assessing pellet quality based solely on this parameter is feasible only under specific, consistent experimental conditions. Therefore, when comparing research results that involve this parameter, it is essential to account for the influence of experimental conditions, with applied pressure as a primary factor.

Statistical analysis showed that permanent strain energy had the strongest effect on pellet quality. Permanent deformation energy and the pressure-strain curve profile represent promising areas for studying compaction’s impact on pellet quality.

At elevated moisture levels, a purely linear elastic response was observed. Our findings indicate that as moisture content increases, the threshold pressure for pure elastic response noticeably decreases. For dry materials, pure elastic recovery is likely attributed to the elasticity of particles of the solid phase. In contrast, for moist materials, elastic recovery increases with water content, which can be attributed to the incompressibility of water. These observations, derived from load/unload curve profiles, warrant further analysis and can contribute to the development of a new densification model that accounts for the role of incompressible water.

## Figures and Tables

**Table 1:** Multivariate analysis of variance by three-way ANOVA of elastic recovery ER_in-die_.

Effect	SS	DF	MS	F	*p*
Intercept	420,531.5	1	420,531.5	4,107,579	*p* < 0.0001
Moisture	1156.9	4	289.2	2825	*p* < 0.0001
Cycle	2410.4	9	267.8	2616	*p* < 0.0001
Pressure	17,697.2	4	4424.3	43,215	*p* < 0.0001
Moisture × cycle	266.9	36	7.4	72	*p* < 0.0001
Moisture × pressure	65.8	16	4.1	40	*p* < 0.0001
Cycle × pressure	4.2	36	0.1	1	0.2633
Moisture × cycle × pressure	9.9	144	0.1	1	0.9979
Error	51.2	500	0.1		

SS—sum of square, MS—mean sum of square, DF—degree of freedom, F—F value.

**Table 2:** Multivariate analysis of variance by three-way ANOVA of non-recoverable energy *E_n-r_* _2–10_ (without cycle 1).

Effect	SS	DF	MS	F	*p*
Intercept	4728.1	1	4728.1	941,654.8	*p* < 0.0001
Pressure	173.2	4	43.3	8627.4	*p* < 0.0001
Moisture	48.8	4	12.2	2430.3	*p* < 0.0001
Cycle	389.6	8	48.7	9700.2	*p* < 0.0001
Pressure × moisture	37.2	16	2.32	463.4	*p* < 0.0001
Pressure × cycle	10.3	32	0.32	64.7	*p* < 0.0001
Moisture × cycle	1.58	32	0.049	9.85	*p* < 0.0001
Pressure × moisture × cycle	3.24	128	0.025	5.04	*p* < 0.0001
Error	2.25	1	0.005		*p* < 0.0001

SS—sum of square, MS—mean sum of square, DF—degree of freedom, F—F value.

**Table 3:** Correlation matrix between some densification variables.

Parameter	SI	ER_in-die_	E_n-r_	%E_n-r_	E_r_	%E_r_	PD
SI							
ER_in-die_	0.142						
E_n-r_	0.646	0.752					
%E_n-r_	−0.221	−0.925	−0.654				
E_r_	0.395	0.954	0.837	−0.916			
%E_r_	0.221	0.925	0.654	−1.000	0.916		
PD	−0.026	0.956	0.699	−0.826	0.845	0.826	

SI—strength index of a pellet, ER_in-die_—elastic recovery, E_n-r_—non-recoverable energy, %E_n-r_—share of non-recoverable energy, E_r_—elastic (dissipated) energy, %E_r_—share of the elastic (dissipated) energy, PD—pellet density.

## Data Availability

The original contributions presented in the study are included in the article, further inquiries can be directed to the corresponding authors.
